# Phenylboronic Acid-Grafted Chitosan Nanocapsules for Effective Delivery and Controllable Release of Natural Antioxidants: Olive Oil and Hydroxytyrosol

**DOI:** 10.3390/pharmaceutics15010081

**Published:** 2022-12-27

**Authors:** Omnia M. Hendawy, Mohammad M. Al-Sanea, Rehab M. Elbargisy, Hidayat Ur Rahman, Ahmed A. B. Mohamed, Islam Kamal, Reda F. M. Elshaarawy, Amgad I. M. Khedr, Wesam Abd El-Fattah

**Affiliations:** 1 Department of Pharmacology, College of Pharmacy, Jouf University, Sakaka 72341, Saudi Arabia; 2 Department of Pharmaceutical Chemistry, College of Pharmacy, Jouf University, Sakaka 72341, Saudi Arabia; 3 Department of Pharmaceutics, College of Pharmacy, Jouf University, Sakaka 72341, Saudi Arabia; 4 Department of Clinical Pharmacy, College of Pharmacy, Jouf University, Sakaka 72341, Saudi Arabia; 5 Department of Chemistry, College of Science, Jouf University, Sakaka 72341, Saudi Arabia; 6 Department of Pharmaceutics, Faculty of Pharmacy, Port Said University, Port Said 42526, Egypt; 7 Department of Chemistry, Faculty of Science, Suez University, Suez 43533, Egypt; 8 Institute of Inorganic Chemistry and Structural Chemistry, Heinrich-Heine University Dusseldorf, 40225 Dusseldorf, Germany; 9 Department of Pharmacognosy, Faculty of Pharmacy, Port Said University, Port Said 42526, Egypt; 10 Chemistry Department, College of Science, IMSIU (Imam Mohammad Ibn Saud Islamic University), Riyadh 11432, Saudi Arabia; 11 Department of Chemistry, Faculty of Science, Port Said University, Port Said 42526, Egypt

**Keywords:** virgin olive oil and hydroxytyrosol, phenylboronic acid-chitosan nanocapsules, pharmaceutical characteristics and potentials

## Abstract

Olives and virgin olive oil (VOO) are a staple of Mediterranean diets and are rich in several beneficial phenolic compounds, including hydroxytyrosol (HT). Therefore, VOO was extracted from Koroneiki olive fruits, and its volatile as well as phenolic components were identified. Meanwhile, in order to upgrade the pharmaceutical capabilities of VOO and HT, a new conjugate phenylboronic acid-chitosan nanoparticles (PBA-CSNPs, NF-1) was fabricated and applied as nanocapsules for implanting high loading and efficient delivery of VOO and HT nanoformulations (NF-2 and NF-3). Due to the H-bonding interactions and boronate ester formation between the hydroxyl groups of the phenolic content of VOO or HT and the PBA groups in the nanocapsules (NF-1), VOO and HT were successfully loaded into the PBA-CSNPs nanocapsules with high loading contents and encapsulation efficacies. The NF-2 and NF-3 nanoformulations demonstrated physicochemical stability, as revealed by their respective zeta potential values, and pH-triggered drug release characteristics. The in vitro studies demonstrated that the nascent nanocapsules were almost completely nontoxic to both healthy and cancer cells, whereas VOO-loaded (NF-2) and HT-loaded nanocapsules (NF-3) showed efficient anti-breast cancer efficiencies. In addition, the antimicrobial and antioxidant potentials of VOO and HT were significantly improved after nanoencapsulation.

## 1. Introduction

The availability, cost-effectiveness, and lack of adverse effects of natural antioxidants have piqued the curiosity of numerous pharmaceutical researchers [1,2]. Essential oils (EOs) derived from plants, spices, and culinary herbs have long been recognized as renewable sources for natural antioxidants because they contain an abundance of polyphenols, carotenoids, and vitamins that have significant levels of antioxidant activity [3,4]. An essential oil’s antioxidant properties are of enormous interest in the pharmaceutical, cosmetic, food, and other industries [3,4]. Among EOs, the exceptional organoleptic, biological, and nutritional quality of virgin olive oil (VOO), obtained from the *Olea europaea* species, has made it an integral part of the Mediterranean diet [5]. Furthermore, VOO has been labeled as a powerful natural antioxidant due to the presence of a variety of different forms of strong antioxidants among its compounds [5,6]. The major antioxidants identified and quantified in VOO are phenolic compounds which can be divided either into lipophilic and hydrophilic phenols, or into five classes, namely: simple phenols (hydroxytyrosol, tyrosol, 2,6-Di-tert-butylhydroquinone (DTBHQ)); phenolic acids (vanillic acid, gallic acid); secoiridoids (oleuropein, aglycone of ligstroside, and their derivatives); flavones (luteolin); and lignans ((+)-pinoresinol and (+)-1-acetoxypinoresinol) [5,6]. Each of these five categories exhibit powerful antioxidant qualities. In addition, a high intake of extra-virgin olive oil, which is abundant in phenolic antioxidants (in addition to squalene and oleic acid), may provide substantial protection against cancer, cardiovascular disease, and aging by reducing oxidative stress [7]. However, the pharmaceutical applications of VOO are still restricted due to its poor stability, oxidative and heat stress as well as the poor absorption and low bioavailability of its active compounds [8,9].

Hydroxytyrosol (HT), the strongest bioactive and antioxidant compound among VOO compounds [10], has garnered a great deal of attention as a natural antioxidants due to its diverse pharmacological effects on numerous diseases, such as inflammatory diseases [11], cardiovascular diseases [12], autoimmune diseases [13], central nervous system disorders [14], etc. Furthermore, HT has been shown to have a variety of biological properties, including those of an aphrodisiac, antidepressant, antimicrobial, anticancer, and antioxidant [15,16]. However, the pharmaceutical applications of HT are still restricted due to its poor stability, oxidative and heat stress, poor absorption, and low bioavailability as evident from post-intake pharmacokinetic analysis [15,17]. Nonetheless, new approaches to the targeted and augmented delivery of HT and VOO have the potential to improve their efficacies. Among these approaches, nanoencapsulation could offer a promising tool to shield this valuable pharmacological agent from the harsh conditions of the stomach and the microbes in the intestines, resulting in greater bioavailability [18,19,20]. It has been recently reported that emulsion systems [21,22], sodium bisulfate cross-linked chitosan [23], liposomes [24], nanogels [18], poly lactide-co-glycolide-co-polyacrylic acid nanoparticles [20], and ethyl cellulose microparticles [25] can effectively encapsulate and improve the bioavailability and biological potentials of HT.

The current study proposes using a phenylboronic acid-chitosan nanoparticle conjugate (PBA-CSNPs) as a nanovehicle to deliver VOO and HT, separately, intracellularly. Because of their optimized drug loading, releasing properties, low toxicity, biodegradability, stability, and renewable nature, biopolymeric nanoparticles (BPNPs) have attracted considerable interest as potential candidates for the controlled delivery of pharmaceutical agents into their targets [26,27,28,29]. Their tiny size and large specific surface improve payload encapsulation stability in the NPs network until the medication release mechanism is initiated and bioavailability is improved as well. Here, the superior anticancer efficacy [30] and high loading capacity [31,32] of the phenylboronic acid-chitosan conjugate will be exploited in the effective loading, efficient delivery, and controlled releasing of VOO and HT, separately, through the nanoencapsulation technique. The nanocapsule-mediated HT delivery formulation has been tested on in vitro breast and lung carcinoma models. Furthermore, the antioxidant performance of new nanoformulations (NFs) (PBA-CSNPs, PBA-CSNPs-VOO, PBA-CSNPs-HT) has been evaluated using 1,1-diphenyl-2-picrylhydrazyl radical scavenging activity (DPPH-RSA) protocol.

## 2. Materials and Methods

The specifications and suppliers of chemicals and solvents used in this work can be found in detail in the electronic Supplementary Material (ESM†). The squid pens chitin and ultrasound-assisted deacetylated chitosan (UCS) were obtained from the previous work [33].

The structures of the new materials were elucidated based upon spectral analyses and physical measurements. The details relating to the instruments used to perform these analyses are also given in the ESM†.

### 2.1. VOO Extraction and Characterization 

#### 2.1.1. Sample Collection

In the fall of 2019, ripe Koroneiki olives were picked from a field in Nubaria, Egypt. To keep the fruits fresh until they were needed, they were placed in dark bottles and chilled to 4 °C. 

#### 2.1.2. Oil Extraction

The olives were crushed in a mixer and centrifuged in a basket using an Abencor laboratory oil mill. Fruits used in the processing were only those that had no signs of sickness or damage. After harvesting, the fresh olive fruits were washed, dried in air, and then crushed at 3000 rpm using a hammer crusher. To separate the oil and pomace from the paste, the crushed fruits were first combined in a mixer at 14 rpm and 25 °C for 60 min, and then centrifuged in a two-phase centrifugal decanter operating at 3500 rpm [34]. Finally, a horizontal centrifuge operating at 6500 rpm and 40 °C was used to extract any remaining suspended materials from the oil. Once the oil samples had been filtered through anhydrous Na_2_SO_4_, they were placed in dark glass vials and frozen at −18 °C until analysis or use.

#### 2.1.3. GC-MS Analysis

Solid phase microextraction–gas chromatography–mass spectrometry (SPME/GC/MS) was used to analyze the extracted VOO for its chemical composition with a Varian 4000 GC/MS mass spectrometer [35]. The oil samples were fractionated using a VF 5ms capillary column (60 μm × 0.25 mm ID, 0.25 μm film thickness). The working conditions were as follows: He gas flow rate of 1.5 mL/min; injection volume of 1.0 L; split ratio of 50:1. The column was kept at 40 degrees Celsius for 10 min, then heated to 180 degrees Celsius at a rate of 20 °C/min, and finally heated to 220 degrees Celsius at a rate of 10 °C/min; temperatures of 250 °C, 270 °C, and 200 °C were used for the injector, transfer line, and ion source, respectively; twenty minutes at 40 °C for pre-incubation; five minutes for desorption. Data were averaged from three biological replicates from three separate studies. At an ionization energy of 70 eV, 2 scans/s, electron impact mass spectra (EI-MS) were acquired. The chemical components of the VOO were specified by comparing their respective retention times and MS patterns to those of standard compounds recorded in the Wiley MS collection 6th edition. VOO compounds were quantified according to the area of their respective peaks in the GC chromatogram.

#### 2.1.4. Phenolic Compound Profiles

The phenolic fraction was recovered from VOO using the previously reported liquid–liquid extraction protocol [36], by combining the oil with an 80/20 *v*/*v* methanol/water solution. After that, the phenolic extract components were analyzed qualitatively and quantitatively using HPLC in accordance with the method described by Selvaggini et al. [37]. Before injecting the phenolic extract into the HPLC, it was diluted in 1 mL of methanol (HPLC quality) and filtered using a polyvinylidene fluoride (PVDF) syringe filter (0.2 µm), to remove any contaminants. The purified extract was then subjected to HPLC analysis utilizing an Agilent Technologies system model 1100 (Agilent Technologies, Palo Alto, CA, USA). A mixed solvents system composed of an aqueous 0.2% acetic acid (solvent A) and methanol (solvent B), with gradient concentrations, was employed as a mobile phase at a flow rate of 1 mL/min. The gradient (A/B) was modified as follows: starts from 95/5 for 2 min, 75/25 for 8 min, 6/40 for 10 min, 50/50 for 16 min, to 0/100 for 14 min. To accomplish equilibration, each composition was kept for 10 min before being reset to the initial state (13 min). Overall, the single analysis took 73 min to run. The diode array detector (DAD) with a wavelength of 278 nm was used to detect secoiridoid and phenolic compound derivatives. The data were represented in mg of phenolic compounds per kg of oil. The standard (reference) phenolic compounds used in this estimation protocol are: gallic acid, ferulic acid, vanillic acid, *p*-salicylic acid, *p*-coumaric acid, syringic acid, vanillin, pyrogallol, coumarin, and caffeic acid (Sigma-Aldrich, Steinheim, Germany); chlorogenic acid, hydroxytyrosol, catechein, salycilic acid, oleuropein, oleuropein glycoside, and epicatechein (Roth, Karlsruhe, Germany); and hydroxytyrosol acetate (VWR international, LLC, Radnor, PA, USA), tyrosol (Janssen Chimica, Geel, Belgium).

### 2.2. Synthesis of PBA-CSNPs Conjugate (NF-1)

Initially, the native CSNPs were prepared according to our group’s earlier study [28]. In brief, a solution of the UCS sample was prepared by solubilizing 0.40 g of UCS in 40 mL of Milli-Q H_2_O (MQW) acidified with glacial acetic acid (0.40 mL) under vigorous stirring at room temperature (RT). After that, the obtained solution was heated to 40 °C and kept at this temperature for 24 h while stirring to get a homogenous hydrogel. After cooling the gelatinous solution to RT, it was subjected to centrifugation at 8500 rpm for 30 min to get rid of the insoluble substance. The gelatinous solution was then dropwise added to a 40 mL aqueous TPP solution (0.2% *w*/*v*) while vigorously swirling, and the entire content was agitated in an ice bath for a further 1 h. Centrifugation at 8500 rpm at 4 °C for 30 min was used to collect the generated CSNPs, which were then washed with MQW (3 × 5 mL). These nanoparticles were subsequently re-dispersed in 40 mL of MQW under ultrasonic irradiation in an ice bath for 10 min to create a homogenous suspension. The suspension was then freeze-dried at −35 °C for 72 h to create CSNPs, which were then kept at −20 °C for subsequent analysis and experiments.

The CSNPs were conjugated with PBA according to a modified method in the previous literature [30]. PBA (33.0 mg, 0.18 mmol) was activated by EDC·HCl (34.6 mg, 0.18 mmol) and NHS (20.6 mg, 0.18 mmol) in dimethyl sulfoxide (DMSO, 2.5 mL) for 2 h, and then was added into the aqueous suspension of CSNPs (5 mg mL^−1^, 6 mL). Following stirring at 37 °C for 24 h, the obtained conjugate was centrifuged at 1000 rpm. The precipitate was washed with ethyl alcohol and deionized water successively to remove unreacted molecules. Next, the PBA-CSNPs conjugate was collected by lyophilization. Finally, PBA-CSNPs conjugate was purified by dialysis for 48 h against MQW using a dialysis bag (MWCO 14 kDa).

### 2.3. Preparation of VOO-Loaded PBA-CSNPs (NF-2)

An aliquot of 0.40 g of NF-1 was dispersed in 40 mL of MQW at RT under ultrasonic irradiation for 30 min, and the content was then stirred at 40 °C for 60 min to ensure homogenization. Then, 0.50 mL of Tween 80 was added to the NF-1 dispersion while stirring at 40 °C, and the obtained mixture was stirred at the same conditions for an additional 2 h to ensure homogenization. To load the oil, 1.0 mL of VOO was solubilized with 8 mL of dichloromethane (DCM) by stirring at RT to obtain a homogenous solution. The oil solution was then added dropwise to the NF-1 dispersion under vigorous stirring at RT. Afterward, the mixture was kept under stirring at RT for an additional 24 h in the dark to ensure homogenization. The obtained nanoformulation (PBA-CSNPs-VOO, NF-2) was collected by centrifugation (8500 rpm) of the content at 4 °C for 30 min and then washed with 5 mL of MQW triplicate. Following that, NF-2 was re-dispersed in 40 mL of MQW for 10 min in an ice bath mediated by ultrasonic irradiation to create a homogenous suspension. Finally, this suspension was subjected to the freeze drying process for 72 h at 35 °C to get pure NF-2, which was stored at −20 °C until use.

### 2.4. Preparation of HT-Loaded PBA-CSNPs (NF-3)

The HT-loaded PBA-CSNPs (NF-3) was prepared as follows: HT (10 mg) and PBA-CSNPs (30 mg) were adequately dispersed in anhydrous DMSO (30 mL). After stirring the mixture for 24 h at RT in the dark, the PBA-CSNPs-HT nanocomposite was collected by centrifuging at 10^4^ rpm for 10 min and then rinsed with phosphate buffered saline (PBS) to remove the unloaded HT, which was determined in the supernatant according to the UV–Vis spectroscopy result. The actually loaded HT was the difference in value between the feeding HT and the unloaded HT in the supernatant. Finally, the HT-loaded nanocapsules (PBA-CSNPs-HT) were re-dispersed in MQW (20 mL) under ultrasonic irradiation (40 kHz) for 10 min in an ice bath, and the dispersion was then frozen to −18 °C before being freeze-dried.

### 2.5. Encapsulation Efficiency and Loading Capacity

Encapsulation efficiencies (EE%) of PBA-CSNPs for VOO in NF-2 and HT in NF-3 were determined by UV–Vis spectrophotometric measurements for VOO and HT at their respective characteristic wavelength (280 nm). An aliquot of 30 mg of nanoformulation (NF-2 or NF-3) was suspended in 30 mL of aqueous ethanol (10%) and then, the suspension was subjected to ultrasonic irradiation (35 kHz) for 1 h. After that, the suspension was centrifuged at 8500 rpm for 10 min to allow freed PBA-CSNPs to settle after the release of VOO or HT to the ethanolic solution. Afterward, to estimate the VOO and HT content (mg/mL) in their respective nanoformulations, the supernatant from each experiment was analyzed spectrophotometrically by measuring the absorbance values at 280 nm. The findings of each experiment were expressed as mean ± SD, with each experiment being performed in triplicate. The value of EE was quantified using Equation (1) [28]:

(1) $$\mathrm{EE}\%=\frac{m_{e}}{m_{f}}\times 100\;$$

where m_e_ and m_f_ are the amounts of encapsulated and feed VOO or HT, respectively. On the other hand, the loading efficiency (LC%) of PBA-CSNPs for VOO or HT was computed using Equation (2) [28]:

(2) $$\mathrm{LC}\%=\frac{m_{e}}{W_{\mathrm{NPs}}}\times 100\;$$

where m_e_ is the amount of encapsulated VOO or HT and W_NPs_ is the weight of PBA-CSNPs.

### 2.6. In Vitro Release

The in vitro release of VOO from NF-2 and HT from NF-3 was investigated under physiological conditions (pH 7.4 and 37 °C) using PBS as a releasing medium following a protocol modified from the previous studies [18,38]. In brief, 20 mL of PBS was added to 2 mg of nanocomposite into a glass vial and the content was incubated in the dark for 48 h at 37 °C with sporadic shaking. Sampling for the release of VOO/HT was done periodically and then centrifuged at 10^4^ rpm to remove suspended materials before being analyzed spectrophotometrically at 280 nm for estimation of the VOO or HT content. The PBS medium was replenished with the same quantity of fresh PBS after each sampling. The cumulative release of VOO/HT from NCs was computed using Equation (3):

(3) $$\mathrm{Release}\left(\%\right)=\overset{t}{\underset{t=0}{\sum }}\frac{M_{t}}{M_{0}}\times 100\;$$

where M_0_ and M_t_ are the amounts of initial and released VOO/HT at each sampling time, respectively. The findings of three independent replicates were used and presented as the mean ± SD.

### 2.7. Antimicrobial Activity

#### 2.7.1. Well Diffusion Assay

Three strains of the serious ESKAPE pathogens [39] (*S. aureus* (SA, ATCC-29737), *P. aeruginosa* (PA, ATCC-27853), and *K. pneumoniae* (KP, ATCC-13883)) were used to investigate the antibacterial potentials of the newly prepared nanocomposites. Amoxicillin (AMX) and tetracycline (TC) antibiotics were used as the “positive” controls in this study. All of the bacterial strains were kindly supplied by NODCAR, Cairo, Egypt, and these strains were routinely cultivated in the nutrient broth agar (NBA). Initially, Mueller Hinton Broth (MHB) was used to inoculate the bacterial suspension at a concentration of 10^6^ CFU/mL and the content was then incubated in a 5% CO_2_ environment at 37 °C for 24 h. Next, the Well diffusion assay (WDA) method described in previous work [40] was used to determine the bacterial strains susceptible to NCs treatments. The inhibition zone diameters (IZD, mm) were used as the key determinants of the NPs’ antibacterial efficacy. Findings of three independent replicates were used and presented as mean ± SD.

#### 2.7.2. Minimal Inhibitory/Bactericidal Concentrations (MIC/MBC)

The antibacterial activity indices, MIC and MBC, of new nanocomposites and clinical antibiotics against tested ESKAPE pathogens were examined using the microtiter assay as outlined previously [41]. In brief, adequate dispersions of NCs and antibiotics in DMSO were prepared and diluted with MHB to prepare the treatment samples at concentrations ranging from 0.25 μg/mL to 50.0 g/mL. Thereafter, 190 μL of bacterial suspensions (10^6^ CFU/mL) were transferred to microtiter plates with 96 wells and then treated with samples of NCs and antibiotics, separately, at the desired concentration. The plates were then incubated at 37 °C for 24 h; untreated wells were used as negative controls. The turbidity of wells was used as indicative of antibacterial efficacies and was used to calculate MIC and MBC values. Findings of three independent replicates were used and presented as mean ± SD.

### 2.8. Antioxidant Activity

#### 2.8.1. 1,1-Diphenyl-2-picrylhydrazyl (DPPH) Radical Scavenging Activity

The antioxidant activities of the formulated nanocomposites (PBA-CSNPs, PBA-CSNPs-VOO, PBA-CSNPs-HT) and their native ingredients VOO, HT, and PBA-CSNPs were investigated using the 2,2-diphenyl-1-picrylhydrazyl radical scavenging activity (DPPH-RSA) method described previously [33]. Briefly, 2.5 mL of an ethanolic DPPH solution (0.1 mM) was added to 0.5 mL of a sample solution in DMSO with serial concentrations (5–320 µg/mL), and the contents were then stirred and kept in a dark environment at 25 °C for 1 h. The DPPH solution served as the experiment’s standard. After keeping the experiment in the dark for 1 h, the absorbance of each sample was measured at 515 nm and the RSA% was computed using Equation (4):

(4) $$\mathrm{RSA}\%=\frac{A_{0}-A_{s}}{A_{0}}\times 100$$

where *A*_0_ and *A*_s_ are the control and sample absorbance values, respectively. Interpolation of the linear regression analysis was used to calculate EC_50_ (μg/mL DPPH).

#### 2.8.2. Lipid Peroxidation Inhibition

Lipoperoxidation inhibition (LPI) was carried out using the procedure outlined by Wright, Colby, and Miles [42]. In brief, 2 mL of a mixture comprising 1 mL of linoleic acid, 0.2 mL of ferric nitrate solution (20 mM), 0.2 mL of ascorbic acid solution (200 mM), and 0.2 mL of hydrogen peroxide (300 mM), was mixed with 100 μL of sample in a vortex shaker. After incubating the reaction mixture for 1 h at 37 °C, the reaction was terminated with 1 mL each of trichloroacetic acid and thiobarbituric acid (10% *w*/*v*). Again, the reaction mixture was incubated for 20 min at 100 °C, and then centrifuged at 5000 rpm for 10 min. Absorbance at 535 nm was measured using a spectrophotometer, and the percentage of inhibition was calculated using the following Equation (5):

(5) $$\mathrm{Inhibition}\%=\left(1-\frac{A_{\mathrm{sample}}}{A_{\mathrm{control}}}\right)\times 100\;$$

### 2.9. In Vitro Anti-Breast Cancer Assay 

#### 2.9.1. Cell Cultures

The cytotoxic effects of the new material were investigated against two cell lines: green monkey kidney (Vero), as representative of normal cells; and breast carcinoma cells (MCF-7). These cells were kindly provided by the American Type Cell Culture Collection (ATCC, Manassas, VA, USA) and cultured in Dulbecco’s Modified Eagle’s Medium (DMEM, Invitrogen/Life Technologies) complemented by fetal bovine serum (FBS, 10%), penicillin (100 U/mL), and streptomycin (100 g/mL) (HyClone, Thermo Scientific). The cells were kept at 37 °C in a CO_2_ incubator (Thermo Scientific Heracell VIOS CO_2_ incubator).

#### 2.9.2. In Vitro Anti-Proliferative Activity

By using the MTT assay, the newly synthesized compounds were evaluated for their cytotoxicity in vitro. Briefly, the cell lines were treated with serial doses (1.56, 3.12, 6.25, 12.5, 25, and 50 µg/mL) of solutions of the tested material in a 96-well plate (10^5^ cells/well) (Falcon, NJ, USA). DMSO and cisplatin (CDDP) were served as negative and positive controls, respectively. After 48 h of incubation at 37 °C in a 5% CO_2_ atmosphere, cells in each well were fixed, rinsed, and stained with an MTT reagent (5 mg/mL in a 0.9% NaCl solution) before being re-incubated for another 4 h. Following incubation, the staining medium was carefully discarded, and 180 μL of acidified isopropanol/well was added to the plate and shaken at room temperature using a MaxQ 2000 plate shaker (Thermo Fisher Scientific Inc., Ann Arbor, MI, USA) to solubilize the produced formazan crystals. The plate was then spectrophotometrically analyzed by measuring absorbance at 570 nm with a Stat FaxR 4200 plate reader (Awareness Technology, Inc., Palm City, FL, USA), to estimate the cell viability.

### 2.10. Statistics

Statistics and computations for this study were completed by a lone student using SPSS v17 for the independent student’s *t*-test and OriginPro 9.1.32 for graphing of results. All results were considered significant if their *P* value was less than 0.05.

## 3. Results and Discussion

### 3.1. Chemical Composition of Volatile Components of VOO

As shown in GC-MS chromatogram (Figure S1, ESM†) and Table 1, 58 major volatile chemical compounds were identified in the extracted VOO. The peak regions on the GC chromatogram for these components account for 99.63% of the entire peak regions. Table 1 shows that the VOO’s main volatile components fall into the following categories: carboxylic and fatty acid esters (accounting for 25.59% of oil content) including limonen-6-ol, pivalate, geranyl acetate, propyl 2-methylvalerate, (E)-2-methyl-tetradecen-1-olacetate (Z)-3-hexenyl acetate, 3,5-Di-t-butyl-1,4-dihydro-phenacetate (major), tetranoic acid ethyl ester, linoleic acid ethyl ester, ethyl iso-allocholate, methyl hexadecadienoate, 2-monoolein, methyl oleate, trielaidin, 1-mono-palmitoylglycerol, and 1-monooleoylglycerol; phenols (accounting for 27.17% of oil content) involving 2,6-Di-tert-butylhydroquinone (major), carvacrol, thymol, 4-allylphenol, pyrogallol, guaiacol, and α-tocopherol; fatty alcohols (accounting for 14.11% of oil content) including 1,9-nonanediol, (E)-3-pentadecen-2-ol, panaxydol, (E)-2-hexen-1-ol, 1-heptatriacotanol, tridecanol, β-sitosterol (major), 1-hexacosanol, and 1-cctacosanol; carboxylic and fatty acids (accounting for 5.87% of oil content) involving pyruvic, oleic (major), 9-octadecenoic, cis-vaccenic, palmitic, linoleic, and stearic; lactones which account for 7.79% of the oil content and involve isochiapin B (major), digitoxin, and tetraneurin-A-diol; other bioactive classes such as aldehydes (2-decenal-(E) being the predominant), terpenes (α-farnesene being the predominant), ketones, (Cholestan-6-one being the predominant) and 1,25-Dihydroxyvitamin D3.

### 3.2. Phenolic Compounds of VOO

Due to their potent antioxidant action and subsequent contribution to shelf-life stability of oil, phenolic compounds are the most significant of the minor compounds in olive oil. Additionally, polyphenols influence the stability and flavor of olive oil and play a significant role in its quality. Therefore, it is important to investigate phenolic compound profiles in the VOO extracted from Koroneiki olive fruits. To that end, the total phenolic compounds were extracted from VOO and their compositions were analyzed by the HPLC-DAD method. The peaks were identified by matching the relative retention times with those of the standard phenolic compounds.

According to the findings in Table S1 (ESM†), twenty phenolic compounds were identified in the composition of Koroneiki VOO: gallic acid, hydroxytyrosol, tyrosol, pyrogallol, p-salycilic acid, vanillic acid, p-coumaric acid, ferulic acid, hydroxytyrosol acetate, catechein, chlorogenic acid, epicatechein, caffeine, caffeic acid, oleuropein, coumarin, ellagic acid, salicylic acid, vanillin, and cinnamic acid. Significantly, the phenolic acids (ferulic, chlorogenic acid, and ellagic), oleuropein, and pyrogallol are the most predominant compounds in the extracted Koroneiki VOO.

### 3.3. Synthesis of PBA-CSNPs Conjugate

**Scheme 1:**
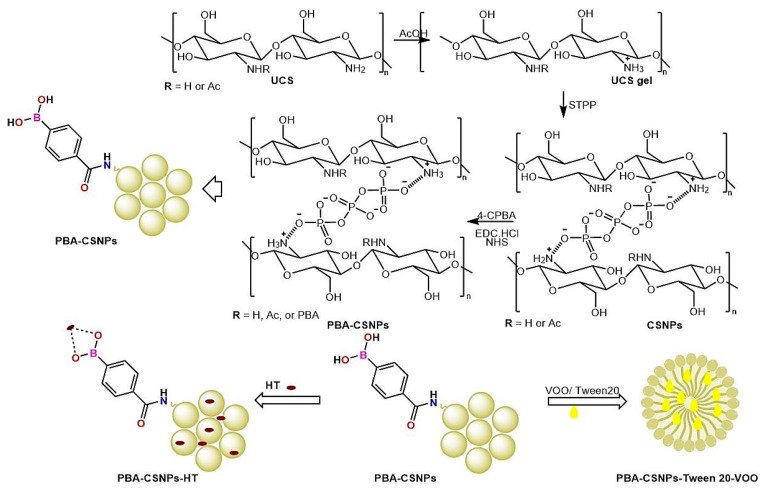
Stepwise synthesis of CSNPs, PBA-CSNPs conjugate and in applications in the synthesis of NFs (PBA-CSNPs-VOO, PBA-CSNPs-HT).

**Figure 1:**
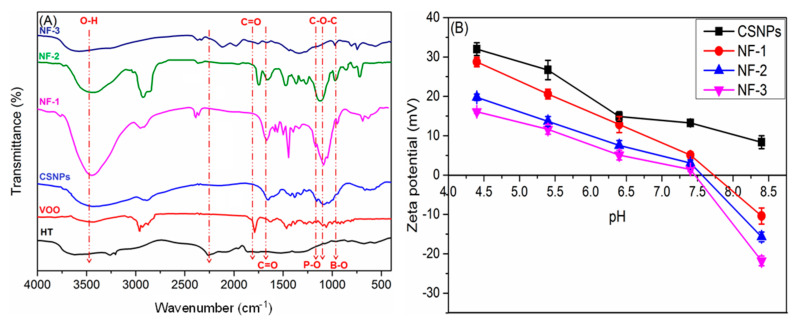
(**A**) FTIR spectra of the native VOO, HT, CSNPs, PBA-CSNPs (NF-1), PBA-CSNPs-VOO (NF-2), and PBA-CSNPs-HT (NF-3). (**B**) Effect of pH on the value of ZP of CSNPs, NF-1, NF-2, and NF-3.

Initially, CSNPs were prepared by the self-assembly of the cationic UCS polyelectrolyte with sodium tripolyphosphate (STPP) as a cross-linking agent, using the ionotropic gelation process [28]. After that, 4-carboxyphenylboronic acid (CPBA) was coupled through amide reaction with the amino groups in UCS chains in the presence of EDC·HCl and NHS, as activating and coupling agent, respectively, to give boronic acid-decorated nanoparticles (PBA-CS NPs), as shown in Scheme 1. To examine the CPBA coupling, the FTIR and energy-dispersive X-ray (EDX) spectra of CPBA, CSNPs and PBA-CSNPs were measured, and shown in Figure 1 and Figure S1 (ESM†). Finally, the PBA-CSNPs-based NFs (PBA-CSNPs-VOO, PBA-CSNPs-HT) were assembled by the nanoencapsulation VOO, or HT into the PBA-CSNPs network, through H-bonding interactions (Scheme 1).

### 3.4. Physicochemical Characterization

The structures of PBA-CSNPs conjugate and its NFs were characterized by FTIR (Figure 1) and EDX analyses.

#### 3.4.1. Elemental Analyses

The molecular weights (*M*_w_) of an UCS sample was estimated from the inherent viscosity ([*η*]) value of its solution in aqueous CH_3_COOH/NaCl solutions at 25 °C according to the Mark–Houwink–Sakurada (MHS) equation: [*η*] = *k*.M^α^ where *k* = 1.81 × 10^−3^ cm^3^/g and α = 0.93. On the other hand, the degrees of acetylation, cross-linking, and coupling (DA, DCL, DC) were computed from the findings of elemental analysis (EA) as outlined in an earlier study [43]. The results of these calculations are collected in Table 2.

#### 3.4.2. FTIR

The main characteristic bands in the region of 3000–3700, 1595, and 1158 cm^−1^ (Figure 1A) correspond to the stretching and bending vibrations of NH/OH, amino, and glycosidic (C-O-C) groups of chitosan, respectively. The emergence of new stretches in the CSNPs spectrum at 1258, 1157, 1089, and 896 cm^−1^ corresponding to the stretching vibrations of PO, PO_2_, PO_3_, and P-O-P moieties of TPP, respectively, is evidence of the successful cross-linking between chitosan and TPP to form CSNPs [28]. After modification with PBA, the C=O stretching vibration of amide I also appears in this region. In addition, the absorptions around 1509 and 851 cm^−1^ are due to the stretching vibration of C=C in the benzene ring and B-O in the carboxyphenylboronic acid group, respectively [30].

The prominent characteristic peaks of the VOO spectrum can be seen at 3449 cm^−1^, assigned to the hydroxyl group of phenolic and alcoholic compounds; 1749 cm^−1^, distinctive of the carbonyl group of esters; 1643 cm^−1^, characteristic of carbonyl group of aldehydes/ketones; and 1333 cm^−1^, for phenolic OH. After VOO loading, three sets of IR spectral peaks distinctive of PBA-CSNPs, VOO, and Tween 20 can be seen in the spectrum of VOO-loaded PBA-CSNPs (NF-2), but with remarkable alterations in their properties due to their mutual interactions. For instance, remarkable shifts (Δν ranging from −16 cm^−1^ to +28 cm^−1^) with or without diminishing intensities were observed in the main VOO peaks (vide supra) due to encapsulation. Meanwhile, the noticeable changes in the amide I, glycoside, P-O, and B-O stretches of PBA-CSNPs are evident of the successful loading of VOO into its matrix. Moreover, a new peak emerged at 1743 cm^−1^ (distinctive of C=O of ester), demonstrating the involvement of Tween 20 in the encapsulation of PBA-CSNPs for VOO (see Figure 1A).

On the other hand, after HT loading, the -OH stretching vibration band is lower than that of PBA-CSNPs and HT, indicating the formation of boronate ester between the B-OH groups in PBA-CSNPs conjugate and -OH groups in HT [30]. 

#### 3.4.3. EDX

The types of elements as well as their contents were also determined by EDX analysis (Figure S2; ESM†). These results further confirmed the successful grafting of PBA on the surface of CSNPs as revealed by the emergence of a new peak in the spectrum of PBA-CSNPs (Figure S2), assignable to the elemental boron. Meanwhile, as shown in the EDX spectrum of PBA-CSNPs-HT, changes in the relative intensities of element peaks and, consequently, the relative percent of elements confirm its successful formation.

#### 3.4.4. Zeta Potential

The surface charge density, suspension’s physical stability, and bioavailability of bioactive material are strongly correlated to its zeta potential (ZP) value. Therefore, the values of ZP for the new bioactive materials were measured and the findings were collected in Figure 1B. When the pH value climbed from 4.5 to 8.5, the ZP of CSNPs reduced from +31.99 mV to +8.59 mV. By contrast, the positive ZP of PBA-CSNPs (28.85 mV) turned into a negative (−8.93 mV) under the same change in pH. This implies that the surface PBA groups on PBA-CSNPs are deprotonated at elevated pH values. The PBA-CSNPs therefore possess the property of reversible surface charge. It is noteworthy that the ZP of CSNPs and PBA-CSNPs are respectively 13.81 mV and 5.93 mV in physiological conditions (pH = 7.4). Along the same lines, the positive values of ZP for VOO-loaded PBA-CSNPs (NF-2) (+16.21 mV) and HT-loaded PBA-CSNPs (NF-3) (+19.76 mV) were turned into negatives of −21.39 mV and −15.13 mV, respectively, by increasing the pH from 4.5 to 8.5. The nanocapsules (NF-2 and NF-3) formed exhibit high zeta potential values, indicating that they are more resistant to agglomerating into bulky particles owing to repulsive interactions [23,44].

### 3.5. Morphological Characterization

#### 3.5.1. SEM Analysis

**Figure 2:**
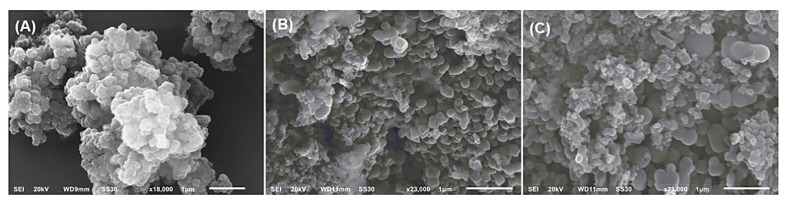
SEM images of different NFs: (**A**) PBA-CSNPs (NF-1), (**B**) PBA-CSNPs-VOO (NF-2), (**C**) PBA-CSNPs-HT (NF-3).

The surface morphologies of the new nanoformulations were investigated using scanning electron microscopy (SEM). The SEM micrograph of NF-1 (Figure 2A) revealed that a large population of semi-spherical nanoparticles are scattered on its surface, which agglomerate into assemblies of a spongy shape. The hydrophilic interactions of the grafted PBA components and swelling of the CSNPs as a result of the stacking of PBA components on their surfaces are two potential causes of the agglomeration of PBA-CSNPs. Because of their porous structure, PBA-CSNPs (NF-1) can effectively encapsulate VOO and HT thanks to their active, accessible cavities. In contrast, the agglomeration propensity of PBA-CSNPs was reduced after the loading of VOO, as shown in Figure 2B. This could be attributed to the formation of a hydrophobic repulsive oil layer on the surface of PBA-CSNPs. These findings agree with the results and hypotheses of Hassan et al.’s work [27]. The aggregation tendency of PBA-CSNPs, on the other hand, was somewhat reduced but still greater than NF-2, and the average size of PBA-CSNPs rose following the loading of HT. This may be explained by the hydrophobic interior of the PBA-CSNPs being occupied by VOO particulates, which forces the hydrophilic HT molecules to the hydrophilic surface of the PBA-CSNPs and increases its average diameter.

#### 3.5.2. Particle Size Distribution (PSD)

**Figure 3:**
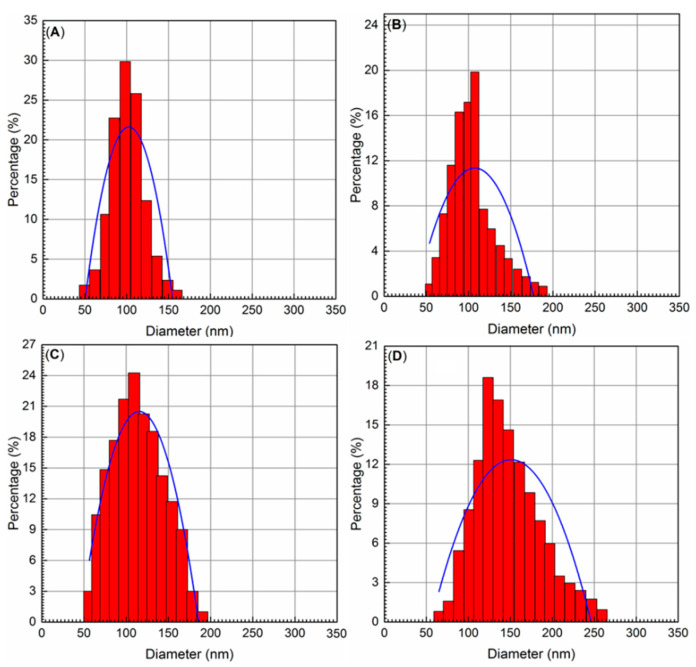
PSD histograms of NFs obtained using different PBA-CSNPs: (**A**) CSNPs, (**B**) PBA-CSNPs, (**C**) PBA-CSNPs-VOO, and (**D**) PBA-CSNPs-HT.

Dynamic light scattering (DLS) was used to investigate the PSD of the native CSNPs and their nanoformulations (NF-1, NF-2, and NF-3). According to Figure 3A, CSNPs had particle diameters (PD) between 49.50 and 149.29 nm and polydispersity indices (PDI) between 0.28 and 0.36. However, the particle sizes of CSNPs were noticeably enlarged after the grafting of PBA on their surfaces, reaching the range of 54.21 nm (PDI 0.28) to 178.14 nm (PDI 0.37) (see Figure 3B). After encapsulation of VOO in NF-2, the PD of PBA-CSNPs was slightly increased into the range of 57.28–190.11 nm with PDI values ranging from 0.32 to 0.37 (see Figure 3C). On the other hand, the PD of PBA-CSNPs in NF-2 (after HT loading) was remarkably increased into the range of 65.13–246.07 nm with PDI values ranging from 0.31 to 0.42 (see Figure 3D). It is noteworthy that low PDI values for CSNPs and their NFs are suggestive of their narrow-size distribution in their respective suspensions, and as a result, a homogenous dispersion is achieved, which is beneficial for pharmaceutical applications.

### 3.6. Packing Properties of New PBA-CSNPs Nanocapsules

As one of the most important pharmaceutical parameters in the encapsulation process, the PBA-CSNPs nanocapsules’ packing properties for encapsulating VOO and HT including encapsulation efficiency (EE) and loading capacity (LC) were evaluated. The packing parameters (EE and LC) of PBA-CSNPs nanocapsules encapsulating VOO (PBA-CSNPs-VOO, NF-2) were quantified as 97.8% and 3.5%, respectively, indicating the promising potential of Tween 20-PBA-CSNPs for loading and encapsulation of Koroneiki VOO and thus essential oils (EOs). Furthermore, the PBA-CSNPs conjugate has better interfacial properties than its native ingredients [30]. The evaporation of the oil’s volatile components during the encapsulation process could be a plausible explanation for the insignificant oil loss (2.2%) of VOO. On the other hand, the estimated EE and LC of PBA-CSNPs nanocapsules encapsulating HT (PBA-CSNPs-HT, NF-3) were found to be 81.25% and 1.6%, respectively. The concentration of HT in NF-3 was significantly higher than in all previously reported HT-based nanocapsules [18,23,25], revealing the promising capabilities of PBA-CSNPs in superior packaging of HT and other natural antioxidant molecules.

### 3.7. In Vitro Release Profiles

The release profile of the bioactive ingredients from the PBA-CSNPs nanocapsules is a crucial pharmacokinetic parameter in the accreditation of the suitability of using PBA-CSNPs as nanocapsules for these ingredients. Therefore, the release styles of VOO-loaded and HT-loaded PBA-CSNPs were investigated under physiological conditions (T = 37 °C, pH = 7.4) by the total immersion method in the releasing media (PBS) for 24 h.

#### 3.7.1. In Vitro OSEO Release Study

**Figure 4:**
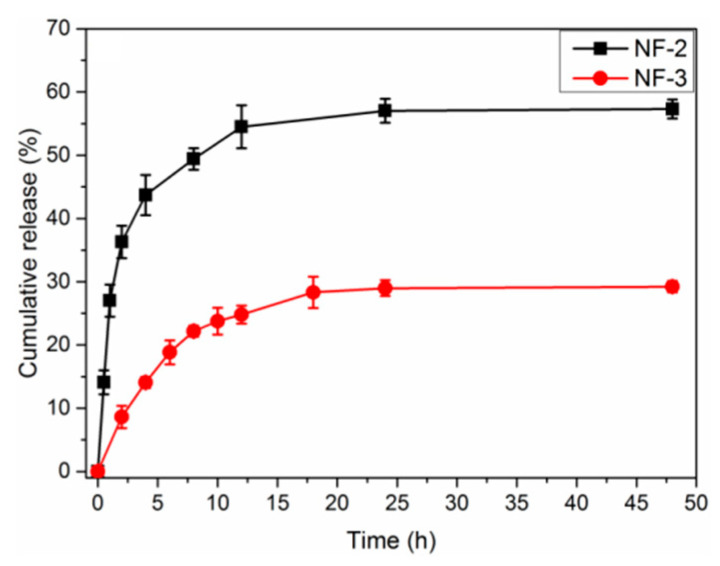
In vitro release profile of the VOO and HT from their respective PBA-CSNPs nanocapsules (NF-2 and NF-3) in PBS solution under physiological conditions.

The VOO release style showed a three-phasic process with an early burst phase, a time of extremely sluggish VOO release rates, and eventually a plateau, with a maximum value of 57% after 24 h (Figure 4). The behavior of the nanocapsule loaded with the drug in the release medium is one of the important parameters influencing drug release in controlled release systems. When the nanocapsule was subjected to the release media during the release studies, the VOO on the inner layers of the nanocapsule began to wash out and somewhat solubilize. Following that, the nanocapsules began to swell, and weight loss ensued. As a result of the high degree of swelling, as well as the solubilization and washing of the nanocapsules, a rapid burst release occurred. Over time, the release medium eventually had enough time to enter the internal layers of the nanocapsules and establish equilibrium [44]. A reduced oil hydrophilia is likely to be the cause of the insignificant release of NF-2 after 24 h of dialysis against PBS.

#### 3.7.2. In Vitro HT Release Study

The encapsulation stability of CS-PBA-HT NF was investigated in PBS at 37 °C (Figure 4). The drug release was slow on the whole, and the burst release in the first 12 h may be attributed to part of HT adsorbed in the surface layer of nanocapsules [18]. The cumulative release of HT within 24 h was about 28%, suggesting that most of the drugs could be stably conjugated on the nanocapsules without ROS presence. The cyclic boronic ester that acts as a ROS-sensitive linker was formed between PBA and hydroxyl groups in HT, which has been widely applied in cancer therapy [18,23].

### 3.8. Antibacterial Assay

**Figure 5:**
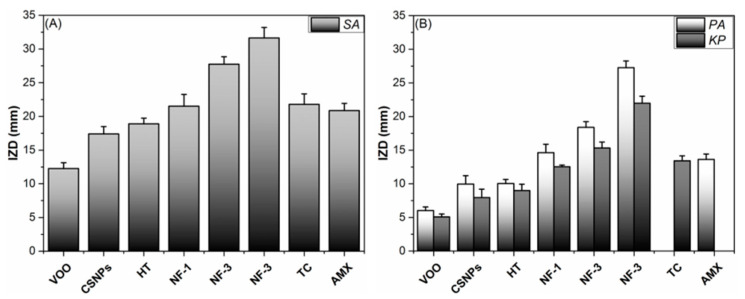
Graph for the inhibition zone diameter (IZD, mm) for the examined NFs against (**A**) Gram-positive bacteria and (**B**) Gram-negative bacteria.

Common foodborne ESKAPE pathogens (SA, PA, and KP) were used to test the in vitro antibacterial efficacy of PBA-CSNPs-mediated synthesized NFs versus Tetracycline (TC) and Gentamicin (GM), two commonly used clinical antibiotics. The sample’s inhibition zone diameter (IZD, mm) was employed as a preliminary antibacterial effectiveness index. Figure 5 demonstrates that all NFs have the potential to inhibit bacterial cell proliferation; nevertheless, their efficacy differs depending on the type of bacterium, the nanoformulation structure, and the size of the NFs themselves. Gram-negative bacterial strains (KP and PA) were generally more resistant to all treatments than Gram-positive strains (SA). This could be due to differences in the structural properties of the two types’ outer bacterial walls, resulting in a variable proclivity of bacterial membrane permeability. Gram-negative bacteria have a more complicated outer membrane than Gram-positive bacteria, which only has a thin peptidoglycan layer, and this extra layer of lipopolysaccharides (LPS), phospholipids (PLs), and lipoproteins (LPs) may serve as a barrier to the entry of antibiotics [45]. It is interesting to note that the IZD values show that when the mean size of NFs decreases, their antibacterial activity increases. For example, NF-3 exhibits the highest antistaphylococcal activity (31.63 ± 1.56 mm). In contrast, the largest NF-1 has the weakest antistaphylococcal activity (17.25 ± 0.87 mm).

Once again, the MIC and MBC values (Table 3) show that Gram-positive bacteria respond better to NFs treatments than Gram-negative bacteria. A possible explanation for this is because its outer surface is covered in negatively charged phosphate groups [46], which interact strongly with the positively charged nanoparticles. Bacterial type and NP size also played major roles in determining the degree of bactericidal or bacteriostatic activities of NFs. For example, the NFs produced by PBA-CSNPs and HT (NF-3) was the most potent antistaphylococcal agent (MIC/MCB = 4.13 ± 0.27/4.27 ± 0.39 μg/mL). On the other hand, the native VOO was the least active antistaphylococcal agent (MIC/MCB = 21.17 ± 1.01/21.72 ± 1.15 μg/mL). It was noteworthy that *K. pneumoniae* is the most resistant bacterial strain for all treatments with MIC/MCB values in the ranging from 22.29 ± 1.11/22.32 ± 0.93 μg/mL to 33.89 ± 1.19/34.13 ± 1.31 μg/mL.

### 3.9. Antioxidant Assay

**Figure 6:**
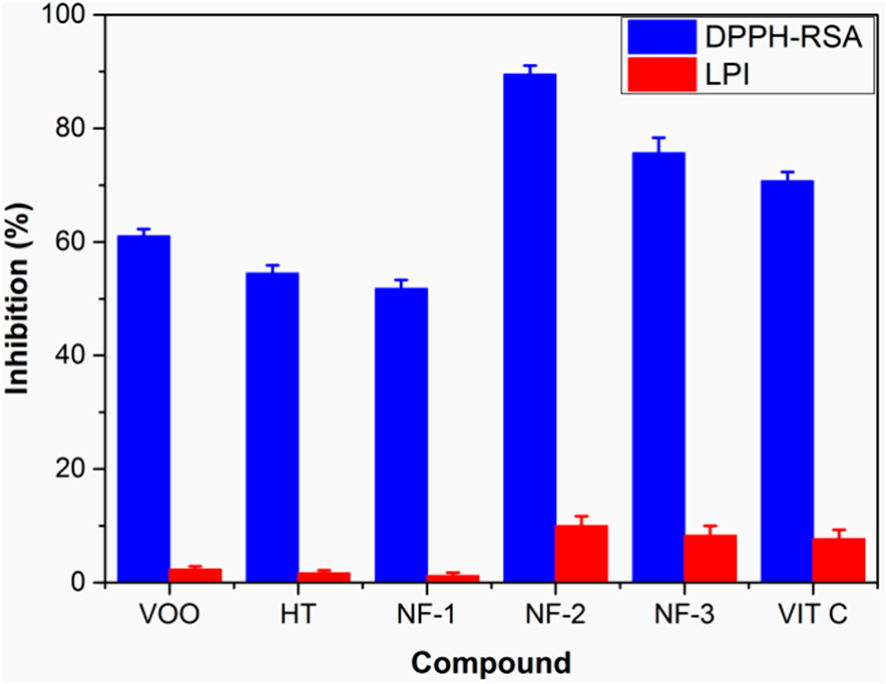
Antioxidant activity (DPPH radical scavenging activity and LPI) of new nanoformulations and their native ingredients.

Figure 6 shows the results of the DPPH-RSA and LPI tests, which evaluate the antioxidant capacity of the nanoencapsulated VOO and HT which were studied in comparison to the bare VOO and HT. The standard antioxidant (Vitamin C, VIT C) was used as a positive control. The findings demonstrated that the DPPH-RSA of VOO-loaded PBA-CSNPs nanocapsules (NF-2) (87.53%) was significantly (*p* ≤ 0.05) higher than both the native nanocapsules (NF-1) (51.78%), the free VOO (61.07%), and VIT C (70.78%). Meanwhile, the anti-radical activity of the HT (DPPH-RSA 54.47%) was significantly (*p* ≤ 0.05) increased after nanoencapsulation by PBA-CSNPs to form NF-3 (DPPH-RSA 75.67%). Overall, our findings support the necessity and significance of the PBA-CSNPs nanocapsules for the delivery of VOO and HT and the achievement of significant antioxidant action at extremely low concentrations.

On the other hand, the LPI monitors the suppression of a prospective chain’s start, propagation, and/or termination of linoleic acid peroxidation in order to assess the free radical mediated antioxidant potential. The free VOO and HT displayed very low LPI values. However, the LPI degree was remarkably enhanced after encapsulation with PBA-CSNPs to reach 9.99% and 8.12% for NF-2 and NF-3, respectively. Overall, these findings support the necessity and significance of the nanoencapsulation technique for delivering VOO and HT and indicate the realizing of significant antioxidant activity at low concentrations.

### 3.10. In Vitro Anti-Breast Cancer Activity

#### 3.10.1. MTT Assay

**Figure 7:**
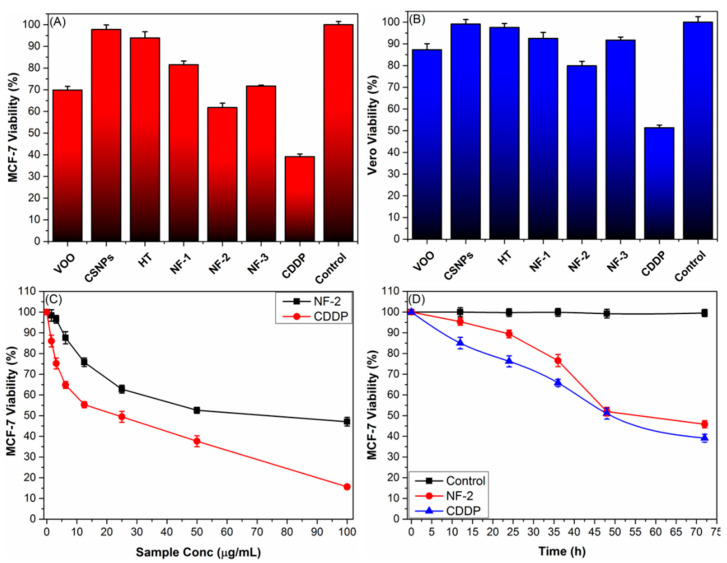
Inhibitory effects of a single dose (25 μg/mL) of PBA-CSNPs-NFs on the proliferative activities of (**A**) MCF-7 cells and (**B**) Vero cells. (**C**) Dose-dependence cytotoxicity performance of the most active anticancer agent (NF-2) against MCF-7, as compared to the clinical anticancer drug (CDDP). (**D**) Time-dependence cytotoxicity performance of IC_50_ dosages of NF-2 and CDDP on MCF-7 cells.

The MTT assay was used to assess the antiproliferative activity of new PBA-CSNPs-coated NFs against cancer (MCF-7) and normal (Vero) cells, with cisplatin (CDDP, 301.1 g/mol) serving as a positive control. When investigating the potential cytotoxic effects of a new drug, it is standard practice to first carry out single-dose tests in human cell lines. The effects of PBA-CSNPs-coated NFs at single treatment doses (25 μg/mL) on the proliferation of MCF-7 cells were therefore investigated (Figure 7A). The MCF-7 cell viability data showed that the NFs derived from the PBA-CSNPs were more efficient in limiting the growth of MCF-7 cells than their native components. Moreover, the NF-2 was the most potent anti-breast cancer agent and could reduce the survival of MCF-7 cells down to 61.75 ± 1.45 at a 25 μg/mL dose. In contrast, the phenylboronic acid-grafted chitosan nanocapsules (NF-1) sample, in comparison, was the weakest anti-breast cancer drug, reducing MCF-7 cell survival to 82.56% ± 1.75 at a 25 μg/mL dose. Additionally, Figure 5B shows that the PBA-CSNPs did not exhibit significant toxicity toward Vero cells even after 48 h of treatment (cell viability > 90%). These findings are in complete agreement with several previous reports that found that NPs’ cytotoxicity is size-dependent, with the smallest ones exhibiting more significant reductions in cancer cell viability [47]. Due to their small size and unique hydrophilic nature, NF-2 are able to diffuse into cancer cells without being taken up by endocytosis and then aggregate inside the cells, where they can cause numerous cytotoxicity processes [47]. Overall, the anti-MCF-7 proliferative activities of PBA-CSNPs-supported NFs are strongly correlated with the structural features of PBA-CSNPs as well as their respective size. Meanwhile, according to the IC_50_ values, VOO-loaded PBA-CSNPs (NF-2) (IC_50_ = 77.29 ± 2.56 μg/mL) exhibited the highest toxicity against MCF-7 and Vero cells, whereas phenylboronic acid-grafted chitosan nanocapsules (NF-1) (IC_50_ = 135.32 ± 2.85 μg/mL) exhibited the lowest cytotoxicity toward MCF-7 and Vero cells. These results demonstrated that chitosan nanocapsules grafted with phenylboronic acid are safe and biocompatible.

#### 3.10.2. Dose- and Duration-Cytotoxicity Correlation

As can be seen in Figure 7C,D, the anti-proliferative activity of the most potent anti-breast cancer agent (NF-2) is significantly impacted by the dose of the chemotherapeutic agent and treatment duration. For example, treatments with 12.5 g/mL and 50 g/mL of NF-2 reduce the number of surviving MCF-7 cells from 100% to 75.8% and 52.2% (24.2% and 47.8% growth reduction), respectively. Cell viability of MCF-7 was drastically decreased after incubation with the IC_50_ dose of NF-3 for 24 h, with the lowest value being seen after 72 h (40.3 ± 1.48%).

## 4. Conclusions

This study was aimed at enhancing the pharmaceutical capabilities (antimicrobial, antioxidant, anti-breast cancer) of the natural antioxidants hydroxytyrosol (HT) and virgin olive oil (VOO) by encapsulating them with biodegradable phenylboronic acid-chitosan nanoparticles (PBA-CSNPs). The VOO was initially extracted from Koroneiki olive fruits, and then its chemical and phenolic constituents were identified using chromatographic analyses. Thereafter, a new PBA-CSNPs conjugate (NF-1) was fabricated and applied as nanocapsules for implanting high loading and efficient delivery of VOO and HT nanoformulations (NF-2 and NF-3). These nanoformulations exhibited superior physiological stability due to H-bonding interactions and boronate ester formation between the OH groups of the phenolic content of VOO or HT and the PBA groups in the nanocapsules. Additionally, in vitro release studies showed that VOO and HT could be released from PBA-CSNPs nanocapsules in a controlled manner under physiological conditions. The nascent—nanocapsules were virtually entirely harmless for both normal and malignant cells. By contrast, the nanoencapsulated VOO-loaded (NF-2) and HT-loaded nanocapsules (NF-3) showed better pharmaceutical capabilities (antimicrobial, antioxidant, anti-breast cancer) compared to the bare VOO and HT. As a result, the PBA-CSNPs nanocarriers may offer a promising safe, biocompatible, and biodegradable nanoplatform for chemotherapeutic applications.

## Figures and Tables

**Table 1:** Chemical composition of VOO.

No.	Compound	RT (min)	Yield (%)	No.	Compound	RT (min)	Yield (%)
1	Limonen-6-ol, pivalate	4.05	2.35	30	Guaiacol	20.82	1.18
2	1,9-nonanediol	4.34	1.41	31	Isochiapin B	20.87	0.89
3	2,6-Di-tert-butylhydroquinone	5.12	15.73	32	Digitoxin	20.99	1.23
4	Pyruvic acid	7.10	1.16	33	1-Heptatriacotanol	21.08	0.80
5	E-3-pentadecen-2-ol	7.78	2.93	34	Tridecanol	21.17	2.63
6	Geranyl acetate	8.46	1.82	35	Linoleic acid ethyl ester	21.25	0.41
7	Carvacrol	8.98	3.77	36	2-Monoolein	21.37	1.23
8	(E)-2-hexenal	9.36	1.30	37	Ethyl iso-allocholate	21.43	1.12
9	Thymol	10.90	1.15	38	Tetraneurin-A-diol	21.75	1.09
10	p-Cymen-7-ol	11.02	1.67	39	β-Sitosterol & α-Farnesene	23.36	3.80
11	Propyl 2-methylvalerate	11.29	0.83	40	Oleic Acid & 9-Octadecenoic acid	23.47	2.53
12	4-Allylphenol	12.21	1.37	41	cis-Vaccenic acid	25.57	1.50
13	(Z)-3-Hexenyl acetate	12.68	0.98	42	Hexadecadienoic acid, methyl ester	27.51	1.28
14	Panaxydol	13.16	0.71	43	Palmitic acid	29.17	0.32
15	E-2-methyl-tetradecen-1-olacetate	13.26	0.73	44	Oleic acid methyl ester	30.25	0.44
16	Pyrogallol	13.45	1.91	45	Linoleic acid	30.95	0.39
17	3,5-Di-t-butyl-1,4-dihydro-phenacetate	13.65	9.24	46	2-Methylenecholestan-3-ol	31.06	1.30
18	Cholestan-6-one	15.88	2.78	47	Stearic acid	32.73	0.36
19	Tetranoic acid ethyl ester	16.75	1.82	48	Trielaidin	34.34	1.01
20	α-N-normethadol	17.02	1.96	49	1,25-Dihydroxyvitamin D3	35.54	3.58
21	Hexanal	17.33	0.82	50	1-Monopalmitoylglycerol	38.47	2.05
22	Heptanal	17.83	1.48	51	1-Mono oleoylglycerol	40.65	0.28
23	Propyl 2-methylvalerate	18.01	0.34	52	Galactinol	41.83	0.29
24	4-nonenal (E)	18.28	1.16	53	Lactitol	42.25	0.41
25	2-decenal-(E)	18.68	1.89	54	Maltitol	42.84	0.31
26	2-decenal-(Z)	18.72	1.04	55	1-Hexacosanol	43.43	0.35
27	10-undecenal	19.68	0.99	56	Hexacosanoic acid	44.65	0.24
28	(E)-2-hexen-1-ol	20.03	1.05	57	α-Tocopherol	45.87	0.29
29	Isochiapin B	20.67	5.81	58	1-Octacosanol	46.0	0.23

**Table 2:** Microanalytical properties of UCS derivatives.

Sample	[*η*] (mL/g)	M_w_ (KDa)	EA (%)			DA	DCL	DC
			C	H	N			
UCS	488.67	691.6	40.14	7.21	7.65	6.2	-	-
CSNPs	-	-	33.43	6.58	5.12	-	11.23	-
PBA-CSNPs	-	-	42.32	7.29	4.87	-	-	32.3

**Table 3:** MIC and MBC values (μg/mL) of new PBA-CSNPs-supported NFs against ESKAPE pathogens, as compared to clinical antibiotics (GM and TC).

Sample	*S* *A*		*PA*		*KB*	
	MIC ± SD	MBC ± SD	MIC ± SD	MBC ± SD	MIC ± SD	MBC ± SD
VOO	21.17 ± 1.01	21.72 ± 1.15	24.56 ± 1.05	25.25 ± 1.25	33.89 ± 1.19	34.13 ± 1.31
CSNPs	15.75 ± 1.12	16.25 ± 1.25	21.07 ± 1.10	21.50 ± 0.95	28.87 ± 1.11	29.05 ± 1.15
HT	14.11 ± 0.85	14.50 ± 0.79	19.85 ± 0.75	20.55 ± 1.03	26.50 ± 1.07	26.75 ± 1.13
NF-1	14.25 ± 0.75	14.77 ± 0.91	18.75 ± 0.89	18.99 ± 0.97	25.97 ± 1.21	26.03 ± 1.25
NF-2	9.95 ± 1.11	10.05 ± 1.13	15.75 ± 0.96	16.15 ± 1.05	23.95 ± 0.99	24.05 ± 1.31
NF-3	4.13 ± 0.27	4.27 ± 0.39	13.56 ± 0.87	14.09 ± 0.98	22.29 ± 1.11	22.32 ± 0.93
GM	NA	NA	16.74 ± 1.25	16.77 ± 1.31	8.27± 0.50	NA
TC	7.20 ± 0.66	NA	NA	NA	9.13± 0.56	NA

NA = not assigned.

## Data Availability

Not applicable.

## Supplementary Materials

The following supporting information can be downloaded at: https://www.mdpi.com/article/10.3390/pharmaceutics15010081/s1, Figure S1: GC-MS chromatogram of virgin olive oil (VOO) extracted from Koroneiki olive fruits; Figure S2: EDX spectrum of PBA-CSNPs; Table S1: Phenolic profile of virgin olive oil (VOO) extracted from Koroneiki olive fruits.
