# Supplementary material for: Phenylboronic Acid-Grafted Chitosan Nanocapsules for Effective Delivery and Controllable Release of Natural Antioxidants: Olive Oil and Hydroxytyrosol
Source: Pharmaceutics. 2022 Dec 27;15(1):81. doi: 10.3390/pharmaceutics15010081 (PMC9867062; doi:10.3390/pharmaceutics15010081)
Supplement: Supplementary file 1 [file pharmaceutics-15-00081-s001.zip › pharmaceutics-2066836-supplementary.pdf]

Phenylboronic acid-grafted chitosan nanocapsules for effective delivery and controllable release of natural antioxidants: Olive oil and hydroxytyrosol

Omnia M. Hendawy[1,*], Mohammad M. Al-Sanea[2], Rehab M. Elbargisy[3], Hidayat Ur Rahman[4], Ahmed A. B. Mohamed[5], Islam Kamal[6], Reda F. M. Elshaarawy, [7,8,*] Amgad I.M. Khedr[9], W. Abd El-Fattah[10,11,]

[1] Department of Pharmacology, College of Pharmacy, Jouf University, Sakaka 72341Saudi Arabia; omhendawy@ju.edu.sa
[2] Department of Pharmaceutical Chemistry, College of Pharmacy, Jouf University, Sakaka 72341, Saudi Arabia; mmalsanea@ju.edu.sa
[3] Department of Pharmaceutics, College of Pharmacy, Jouf University, Sakaka, Aljouf 72341, Saudi Arabia; rmalbargisy@ju.edu.sa
[4] Department of Clinical Pharmacy, College of Pharmacy, Jouf University, Sakaka 72341, Saudi Arabia; hidayat@ju.edu.sa
[5] Department of Chemistry, College of Science, Jouf University, Sakaka 72341, Saudi Arabia; ahmed_smt@yahoo.com
[6] Department of Pharmaceutics, Faculty of Pharmacy, Port Said University, Port Said 42526, Egypt; islamkamal@pharm.psu.edu.eg
[7] Department of Chemistry, Faculty of Science, Suez University, Suez 43533, Egypt
[8] Institute of inorganic Chemistry and structural Chemistry Heinrich-Heine University Dusseldorf, 40225 Dusseldorf, Germany
[9] Department of Pharmacognosy, Faculty of Pharmacy, Port Said University, Port Said42526, Egypt; amged.ibrahim@pharm.psu.edu.eg
[10] Chemistry Department, College of Science, IMSIU (Imam Mohammad Ibn Saud Islamic University), P.O. Box 5701, Riyadh 11432, Saudi Arabia; wabdulfatah@imamu.edu.sa
[11] Department of Chemistry, Faculty of Science, Port Said University, Port Said 42526, Egypt
* Correspondence: omhendawy@ju.edu.sa (O.M.H.); reda.elshaarawy@suezuniv.edu.eg (R.F.M.E.)

*Correspondence: OMH, omhendawy@ju.edu.sa; RFME, reda.elshaarawy@suezuniv.edu.eg,

Contents:

1- **Materials and instrumentation**

2- **Experimental Section**

3- **Modified well diffusion antimicrobial techniques**

4- **Figures**

1. Materials and instrumentation

1.1. Materials

Chemicals were obtained from the following suppliers and used without further purification: hydroxytyrosol (Roth, Germany); 1-(3-dimethylaminopropyl)-3-ethylcarbodiimide hydrochloride (EDC.HCl), N-hydroxysuccinimide (NHS), phenylboronic acid (PBA).

1.2. Instrumentation

Elemental analyses for C, H, N, were performed with a Perkin–Elmer 263 elemental analyzer. FTIR spectra were recorded on a BRUKER Tensor-37 FTIR spectrophotometer in the range 400–4000 cm^{-1} as KBr disc or with an ATR (attenuated total reflection) unit (Platinum ATR-QL, diamond). UV-Vis spectra were measured at 25 °C in ethanol (10^{-5} mol/L) on a Shimadzu UV-2450 spectrophotometer using quartz cuvettes (1 cm). The particle size distribution of the CSNPs and its nanoplatforms was determined by Dynamic light scattering (DLS) and zeta potential in deionized water solution (pH 6.3, ionic strength 0) using a Nicomp 380 ZLS particle seizer (PSS, USA). The morphology of the formed micro and nano-composites was investigated using Scanning electron microscopy (SEM, Hitachi S-7400, Hitachi, Japan) supported with energy dispersive –X-ray (EDX) to determine the elemental analysis of the formed products.

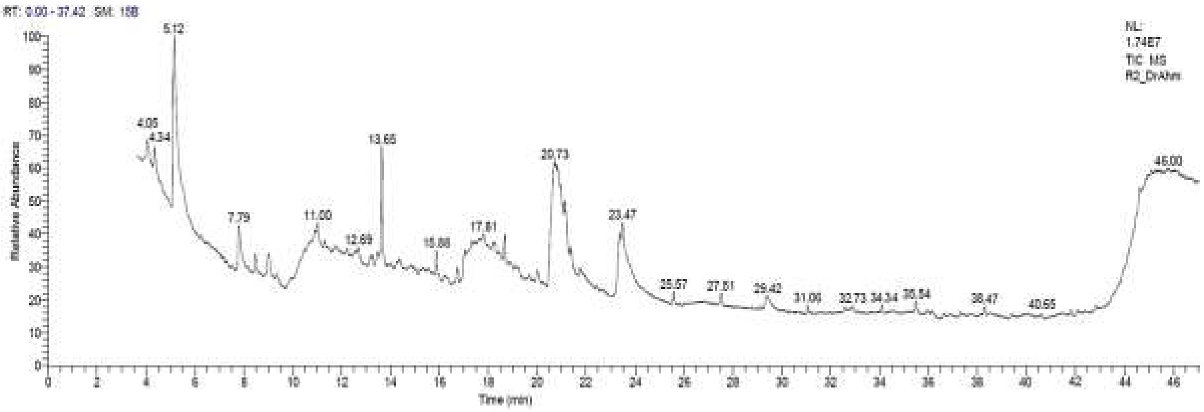

Figure S1: GC-MS chromatogram of virgin olive oil (VOO) extracted from Koroneiki olive fruits

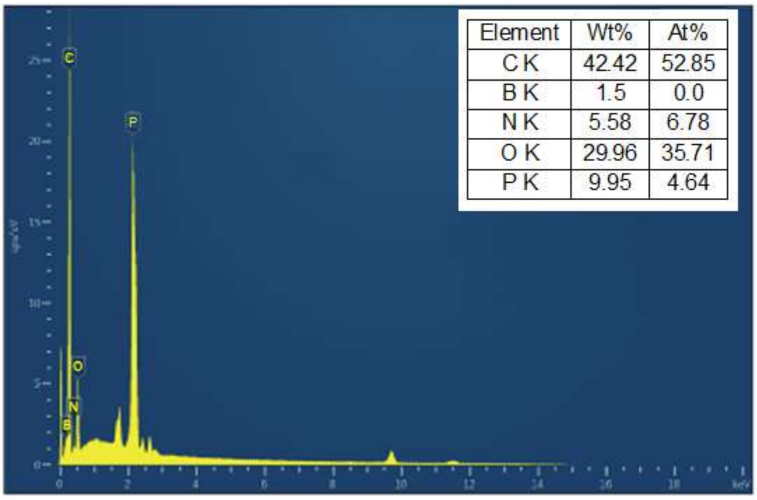

Figure S2: EDX spectrum of PBA-CSNPs

Table S1: Phenolic profilre of virgin olive oil (VOO) extracted from Koroneiki olive fruits

No.	Compound	MF	MW	Yield (%)
1	Gallic acid	$C_7H_6O_5$	170.12	6.05
2	Hydroxytyrosol	$C_8H_{10}O_3$	154.16	5.65
3	Tyrosol	$C_8H_{10}O_2$	138.16	2.82
4	Pyrogallol	$C_6H_6O_3$	126.11	59.75
5	*p*-Salicylic acid	$C_7H_6O_3$	138.12	8.13
6	Vanillic acid	$C_8H_8O_4$	168.15	6.23
7	*p*-Coumaric acid	$C_9H_8O_3$	164.16	3.92
8	Ferulic acid	$C_{10}H_{10}O_4$	194.18	121.13
9	Hydroxytyrosol acetate	$C_{10}H_{12}O_4$	196.20	3.45
10	Catechein	$C_{15}H_{14}O_6$	290.27	134.43
11	Chlorogenic acid	$C_{16}H_{18}O_9$	354.31	125.17
12	Epicatechein	$C_{15}H_{14}O_6$	290.27	1.89
13	Caffeine	$C_8H_{10}N_4O_2$	194.19	1.25
14	Caffeic acid	$C_9H_8O_4$	180.16	2.24
15	Oleuropein	$C_{25}H_{32}O_{13}$	540.52	536.35
16	Coumarin	$C_9H_6O_2$	146.14	135.14
17	Ellagic acid	$C_{14}H_6O_8$	302.20	523.75
18	Salicylic acid	$C_7H_6O_3$	138.12	8.65
19	Vanillin	$C_8H_8O_3$	152.14	3.64
21	Cinnamic acid	$C_9H_8O_2$	148.16	5.81
